# Supplementary material for: Design and Testing of Augmented Reality-Based Fluorescence Imaging Goggle for Intraoperative Imaging-Guided Surgery
Source: Diagnostics (Basel). 2021 May 21;11(6):927. doi: 10.3390/diagnostics11060927 (PMC8224390; doi:10.3390/diagnostics11060927)
Supplement: Supplementary file 1 [file diagnostics-11-00927-s001.zip › diagnostics-1183464.pdf]

Changes in visible-IR FOV correlation

The percentage of matching according to the ICG concentration was calculated and compared. The higher ICG concentration tended to have the higher the percentage of matching. As a result of the experiment, it was confirmed that the correlation was different depending on the ICG concentration, indicating that it was not only dependent on the ICG concentration but also the fluence rates. In order to obtain an accurate fluorescence image at the surgical site, it is necessary to have conditions such as optimal ICG concentration and fluence rate.

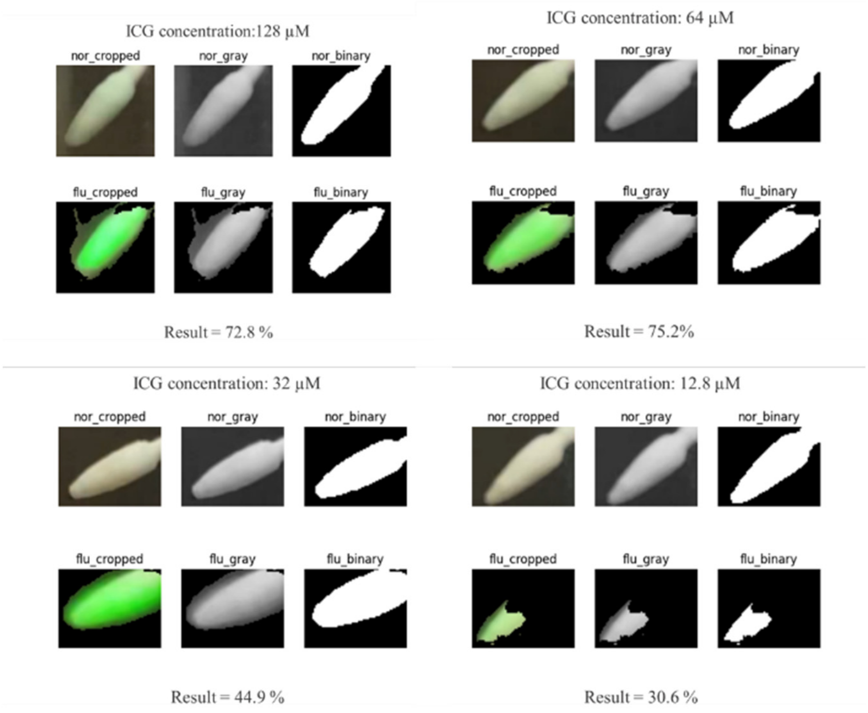

Figure 1. Changes in visible-IR FOV correlation according to ICG concentration

ICG resolution of the system

The actual concentration of ICG used in the clinical field was expected to be 130 µM. Four different concentrations of ICG were used, and the distance between the target and the system was set around 400 mm. As a result, it was confirmed that the fluorescence image excitation from ICG of 16.1 µM at 1/8 level of the expected value (130 µM)was also clearly displayed.

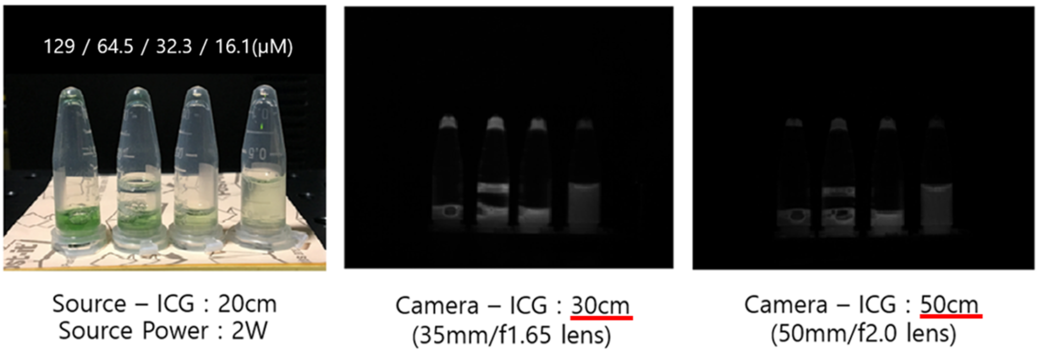

Figure 2. the results of the ICG resolution of the proposed system.
