# Supplementary material for: Design and Testing of Augmented Reality-Based Fluorescence Imaging Goggle for Intraoperative Imaging-Guided Surgery
Source: Diagnostics (Basel). 2021 May 21;11(6):927. doi: 10.3390/diagnostics11060927 (PMC8224390; doi:10.3390/diagnostics11060927)

**Lymphatic Distribution Analysis of ICG in Rabbit Model**

The rabbit subcutaneous tumor model was prepared by injecting VX2 tissues into the subcutaneous tissue of the ankle. At 2–3 weeks after tumor implantation, the tumors were expected to be round with a diameter of approximately 2 cm. The near infrared (NIR) dye ICG was peritumorally injected into the ankle of the rabbit. The distribution of the dye in their lymphatic system was observed and resected under ARFI Goggle guidance.

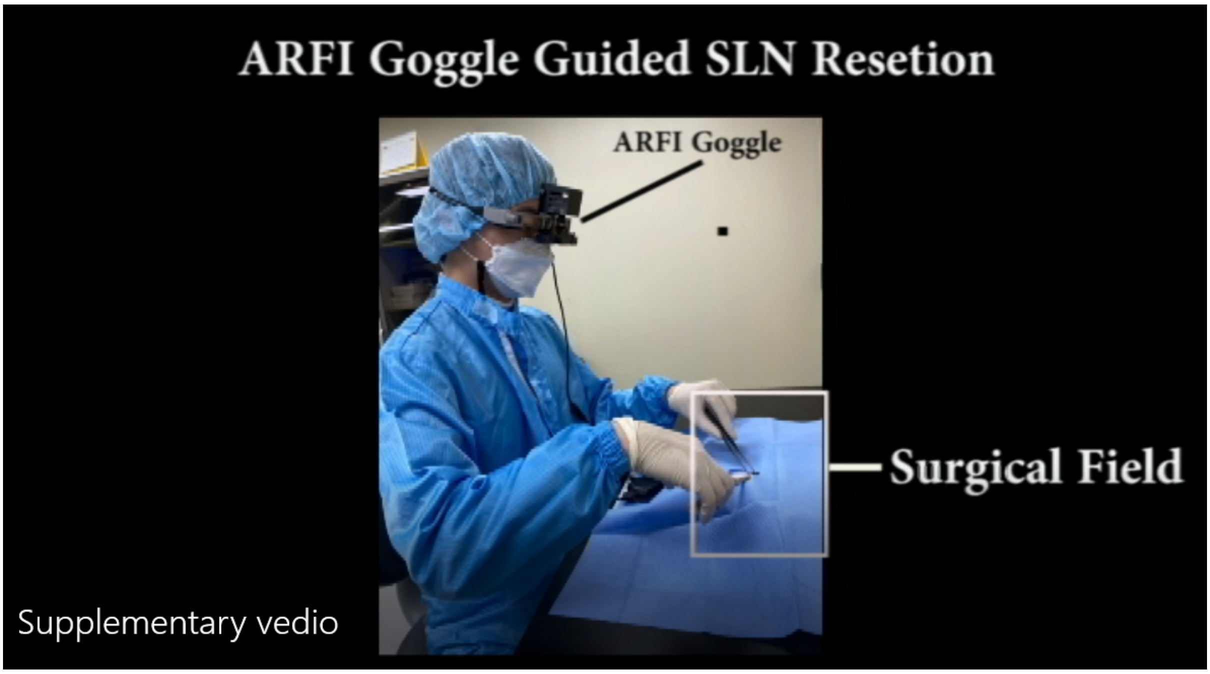

Supplement: Supplementary file 1 [file diagnostics-11-00927-s001.zip › Supplement_vedio.pdf]
